# Association of environmental exposure to perchlorate, nitrate, and thiocyanate with overweight/obesity and central obesity among children and adolescents in the United States of America using data from the National Health and Nutrition Examination Survey (NHANES) 2005–2016

**DOI:** 10.1002/cad.20487

**Published:** 2022-10-17

**Authors:** Qi Jiang, Qin Li

**Affiliations:** ^1^ Department of Pediatric Suining Central Hospital Sichuan China

**Keywords:** central obesity, environmental exposure, National Health and Nutrition Examination Survey, nitrate, obesity, overweight, perchlorate, thiocyanate

## Abstract

The association of overweight/obesity, and central obesity with thiocyanate (SCN), perchlorate (CIO), and nitrate (NO) in childhood and adolescence is unclear. Therefore, this study aimed to explore this association in 4447 participants comprising children and adolescents (aged 6–19 years) using data from the United States National Health and Nutrition Examination Survey 2005–2016. SCN level was positively associated with overweight/obesity in both children and adolescents, while CIO level was negatively associated with overweight/obesity only in children; however, no significant association was found for NO level. Similar associations were found between SCN level and central obesity. Thus, our results suggest that SCN exposure was associated with overweight/obesity and central obesity in both children and adolescents, while a negative association was observed for CIO in children. Strategies to monitor the exposure levels and the mechanisms underlying the relationship between exposure and the weight parameters are recommended.

## 1 INTRODUCTION

The prevalence rates of childhood and adolescent obesity, a serious global public health issue, have been increasing in recent decades (Ng et al., 2014). Globally, 40 million children under the age of 5 years and 330 million children and adolescents aged 5–19 years were either overweight or obese in 2016 (Di et al., 2019). A study including 123113 Greek children indicated that the prevalence of central obesity was 33.4 % (Grigorakis et al., 2016). Furthermore, central obesity may exhibit a stronger correlation with cardiovascular diseases than that exhibited by general obesity (Stefan et al., 2008). Studies have revealed that environmental chemicals are associated with the development of obesity (Holtcamp et al., 2012). Exposures to environmental pollutants in people's lives and at work have been shown to alter their metabolism and disposition of weight gain or loss (Wang et al., 2016).

A previous observational study reported that the early stages of life, including foetal life, infancy, childhood, and adolescence, are critical for the long‐term phenotypic effect of environmental exposures (Rauschert et al., 2017; Wells, 2006). Therefore, weight gain in these stages plays an important role in predicting obesity risk later in life (Simmonds et al., 2016). Adolescents with obesity have a 5‐fold higher risk of developing obesity in adulthood than do adolescents without obesity. Moreover, adolescence is one of the life stages when fat deposition peaks (Wells, 2006), as it is a time of rapid growth when significant changes in the amount and distribution of adipose tissue occur (Mihalopoulos et al., 2010).

Thiocyanate (SCN), perchlorate (CIO), and nitrate (NO), as thyroid disrupting chemicals, can cause thyroid dysfunction, leading to a competitive inhibition of radioactive iodide uptake by the human sodium iodide symporter (Tonacchera et al., 2004). Thyroid hormones are essential for normal growth in children and adolescents, as they promote and modulate growth hormone (GH) secretion (Burstein et al., 1979). People may be exposed to these three chemicals at work and in life through exposure to cigarette smoke; consumption of foods that contain CIO, NO, and SCN; and working in cyanide‐utilising industries, such as those that manufacture furniture and electronic computing equipment (Jain et al., 2016). These three chemicals are metabolised in the blood, urine, saliva, and breast milk in vivo after exposure (Blount et al., 2008; Oldi & Kannan, 2009; Wu et al., 2014; Oluwole, 2013). Several studies used the urinary level of these three chemicals as biomarkers to monitor internal exposure status (Mervish et al., 2016; Liu et al., 2017).

A previous study has shown that higher urinary SCN and CIO levels were associated with a higher risk of iodine deficiency in young children (von Oettingen et al., 2017). A population‐based study from the National Health and Nutrition Examination Survey (NHANES) revealed that exposures to SCN and NO, respectively, were associated positively and negatively with obesity in adults (Zhu et al., 2021). However, the associations between obesity and exposures to SCN, CIO, and NO have not yet been examined in children and adolescents. Therefore, this study aimed to explore the associations of urinary SCN, CIO, and NO levels with the risk of overweight/obesity and central obesity in children and adolescents from the NHANES 2005–2016.

## 2 MATERIALS AND METHODS

### 2.1 Study design and population

This was a cross‐sectional analysis of data from the United States (U.S.) NHANES, which collects data on nutrition and health statuses from a nationally representative sample of non‐institutionalised individuals in the United States, and it was approved by the National Center for Health Statistics (NCHS) Ethics Review Board (Protocol #2005‐06, Protocol #2011‐17). Informed consent was obtained from all participants. All protocols adhered to the principles of the Declaration of Helsinki.

### 2.2 Participants

Our sample included participants aged 6–19 years from the NHANES 2005–2016 who had data on urinary SCN, NO, and CIO. The demographic data included age (children aged 6–11 years; adolescents aged 12–19 years), sex, race (Mexican American/other Hispanic/non‐Hispanic White/non‐Hispanic Black/other races), and ratio of family income to poverty. Ratio of family income to poverty was calculated by dividing family (or individual) income by the poverty guidelines specific to the survey year. The exclusion criteria were as follows: those without data on their usual energy intake, body mass index (BMI), physical activity (assessed for participants aged 12 years and older), urinary creatinine and waist circumstance (WC), individuals who had asthma, thyroid problems, malignancies, liver conditions, kidney conditions, and diabetes, leaving a final sample of 4447 children and adolescents for the analyses.

### 2.3 Measurement of urinary CIO, NO, creatinine, and SCN levels

Ion chromatography coupled with electrospray tandem mass spectrometry was used to quantify SCN, NO, and CIO levels in human urine. To achieve chromatographic separation, ion Pac AS16 column was used with sodium hydroxide as the eluent. An electrospray interface was used to ionise the eluent from the column to generate negative ions and to transport the sample to the mass spectrometer. The lower limits of detection (LOD) for urinary SCN, NO, and CIO levels were 20,700 and 0.05 ng/ml, respectively. The values of LOD divided by the square root of 2 were defined as the measurement levels below the LOD.

### 2.4 Weight status measurement

WC, weight, and height were measured at the mobile examination centres (MEC). BMI was calculated as weight in kilograms (kg) divided by height in meters squared (kg/m^2^) in accordance with the 2000 U.S. Centres for Disease Control and Prevention (CDC) growth charts (Yanovski & Yanovski, 2011). While underweight (designated one) was defined as a BMI value below the age‐ and sex‐specific 5th percentile, normal weight (designated two), overweight (designated three), and obesity (designated four) were defined as BMI values at or above the age‐ and sex‐specific 5th percentile, 85th percentile, and 95th percentile, respectively. Central obesity was defined as WC to height ratio (WtHR) ≥ 0.5 (Lean et al., 2002; Gualdi‐Russo et al., 2015).

### 2.5 Covariate measurement

Urinary creatinine was measured using an enzymatic method, in which creatinine was converted to creatine by creatininase. Then, creatine was broken down by creatinase to form sarcosine and urea. Sarcosine oxidase was used to convert sarcosine to glycine and hydrogen peroxide, and hydrogen peroxide reacts with chromophore in the presence of peroxidase to produce a colour product that is measured at 546 nm (secondary wavelength = 700 nm) (Myers et al., 2006). Physical activity was assessed using the Global Physical Activity Questionnaire; participants were classified as active if they reported more than 10 min of moderate to vigorous physical activity per week (Physical Activity Guidelines Advisory Committee et al., 2021). The dietary interview (individual foods, first day) was used to obtain detailed dietary intake information from the participants based on the U.S. Department of Agriculture Food and Nutrient Database for Dietary Studies 2005−2016 (U.S. Department of Agriculture et al., 2016). Total energy intake was calculated as averages of the 24‐h dietary recall from an interview at MEC.

The NHANES quality assurance and quality control (QA/QC) protocols meet the 1988 Clinical Laboratory Improvement Act mandates.

### 2.6 Statistical analysis

We used the R‐project (http://www.R‐project.org) and EmpowerStats (http://www.Empowerstats.com; X&Y Solutions Inc, Boston MA) to perform all the statistical analyses, and *P* < 0.05 was considered statistically significant. Variables were expressed as mean ± SD or number (%). *P*‐value was calculated by ANOVA for continuous variables and chi‐square test for categorical variables. Covariates were included as potential confounders in the final models if they changed the estimates of these three chemicals in relation to overweight/obesity by more than 10%, if they were associated with overweight/obesity, or if they had clinical significance. In the current cross‐sectional analysis, the urinary levels of CIO, NO×10^3^, and SCN were categorised into quartiles. The full sample 2‐year MEC exam weight (WTMEC2YR) was used for the recalibration, and we constructed three multiple logistic regression models with adjustments for possible baseline data imbalances: model 1: urinary creatinine, age, sex, ratio of family income to poverty, and race were adjusted; model 2: based on model 1, physical activity (age 12−19 years), and the total energy intake were additionally adjusted; model 3: based on model 2, SCN, NO, and CIO levels were adjusted mutually. The categories of the urinary levels of the three chemicals were used in these regression models, and Q1 was used as the reference. We performed tests for a linear trend by entering the quartile values of each urinary level category as a continuous variable in the models and performed smooth curve fitting to examine whether an independent variable was partitioned into intervals. Segmented regression and log‐likelihood ratio tests were conducted to determine whether a threshold existed.

## 3 RESULTS

### 3.1 Characteristics of the study population

The study participants consisted of 2576 children (males, 49%) aged 6–11 years (58%) and 1871 adolescents aged 12–19 years (42%): 29% non‐Hispanic White, 10% other Hispanic, 27% Mexican American, 25% non‐Hispanic Black, and 9% other race. There were more Mexican American children and fewer other Hispanic and other race children. Active physical activity was observed in 39% of the adolescents. The mean ± SD values for urinary SCN, NO × 10^3^, CIO, and creatinine were 1539.76 ± 1989.49 ng/ml, 64.02 ± 54.95 ng/ml, 5.97 ± 9.72 ng/ml and 115.64 ± 72.42 mg/dl, respectively, with higher urinary levels of creatinine and SCN and lower urinary levels of CIO in adolescents than in children. Overall, the proportions of participants who were obese, overweight, of normal weight, and underweight were 21%, 16%, 60%, and 2%, respectively. The proportion of adolescents (36%) with central obesity was higher than that of children (30%) (Table 1).

**TABLE 1:** Baseline characteristics of children (*N* = 2576) and adolescents (*N* = 1871)

	Aged 6–19 years		Aged 6–11 years		Aged 12–19 years		***P*** ^b^
	*N*	Mean ± SD prevalence	*N*	Mean ± SD prevalence	*N*	Mean ± SD prevalence	
Overall, *n*(%)^b^	4447	100%	2576	58%	1871	42%	
Age, y	4447	11.41 ± 3.95	2576	8.48 ± 1.73	1871	15.44 ± 2.24	<0.001
Male^a^, %	2175	49%	1232	48%	948	50%	0.09
Race, %							<0.001
Mexican American	1201	27%	736	29%	465	25%	
Other Hispanic	446	10%	232	9%	214	12%	
Non–Hispanic White	1299	29%	734	28%	565	30%	
Non–Hispanic Black	1096	25%	660	26%	436	23%	
Other race	405	9%	214	8%	191	10%	
Physical activity^c^							
active					728	39%	
inactive					1143	61%	
Ratio of family income to poverty	4447	2.05 ± 1.53	2576	2.03 ± 1.52	1871	2.09 ± 1.55	0.192
Usual energy intake, kcal/d	4447	978.41 ± 578.96	2576	967.29 ± 521.00	1871	993.71 ± 650.20	0.357
CIO (ng/ml)	4447	5.97 ± 9.72	2576	6.34 ± 9.70	1871	5.47 ± 9.72	<0.001
NO×10^3^ (ng/ml)	4447	64.02 ± 54.95	2576	64.74 ± 54.22	1871	63.03 ± 55.93	0.099
SCN (ng/ml)	4447	1539.76 ± 1989.49	2576	1359.61 ± 1328.92	1871	1787.80 ± 2621.51	<0.001
Urinary creatinine (mg/dl)	4447	115.64 ± 72.42	2576	94.60 ± 53.21	1871	144.61 ± 84.38	<0.001
Weight category							0.901
Underweight	104	2%	57	2%	47	3%	
Normal weight	2674	60%	1557	60%	1117	60%	
Overweight	718	16%	413	16%	305	16%	
Obesity	951	21%	549	21%	402	22%	
Central obesity	1443	33%	768	30%	675	36%	<0.001

^a^ Estimated using full sample 2‐year mobile examination centres (MEC) exam weight from the National Health and Nutrition Examination Survey (NHANES).

^b^ *P*‐value calculated using ANOVA for continuous variables and chi‐square test for categorical variables.

^c^ Physical activity were assessed only for participants aged 12 years and older.

Abbreviations: SCN, thiocyanate; CIO, perchlorate; NO, nitrate.

### 3.2 Associations of urinary CIO, NO, and SCN levels with overweight/obesity and central obesity

The results of multiple logistic regression analysis regarding the associations of these three chemicals with overweight/obesity are summarised in Tables 2 and 3. In children, urinary SCN level was associated positively with overweight/obesity. The odds ratio (OR) for overweight/obesity in the highest quartile of urinary SCN compared with that of the lowest quartile was 2.00 (95% confidence interval [CI]:1.45–2.77) (*P* < 0.001), after adjusting for urinary creatinine level, age, sex, ratio of family income to poverty, and race, compared with the reference quartiles (Table 2, Model 1). The positive association became more significant after adjusting for potential misreporting by adding total energy intake, urinary CIO level, and urinary NO level into the model (Table 2, model 3). Similar results were observed in adolescents, and the positive association was more significant than the association among children (Table 3). Urinary CIO level was associated negatively with overweight/obesity only in children, and the OR for overweight/obesity in the highest quartile of urinary CIO level compared with that of the lowest quartile was 0.43 (95% CI: 1.04–2.26) (*P* = 0.03), after adjusting for urinary creatinine level, age, sex, ratio of family income to poverty, race, total energy intake, urinary CIO level, and urinary SCN level, compared with the reference quartiles (Table 2). As shown in Tables 4 and 5, there was a consistent positive association between urinary SCN level and central obesity in children and adolescents, after adjusting for various confounding variables in the models. The trend remained significant among the different urinary SCN level categories in all the models (*P* < 0.001).

**TABLE 2:** Associations between urinary CIO, urinary NO, and urinary SCN and the risk of overweight/obesity in children (*N* = 2576)

Urinary biomarkers	*N*	Model 1	*P*	Model 2	*P*	Model 3	*P*
	2576	OR (95%CI)		OR (95%CI)		OR (95%CI)	
**SCN (ng/**ml)							
Q1 (< 582.75)	644	Ref		Ref		Ref	
Q2 (582.75–1030.00)	632	1.15 (0.86–1.54)	0.33	1.15 (0.86–1.54)	0.34	1.17 (0.88–1.54)	0.29
Q3 (1030.01–1680.00)	648	1.24 (0.92–1.67)	0.16	1.24 (0.92–1.67)	0.16	1.26 (0.93–1.69)	0.14
Q4 (> 1680.00)	652	2.00 (1.45–2.77)	<0.001	2.00 (1.45–2.77)	<0.001	2.05 (1.48–2.84)	<0.001
*P* for trend		<0.001		<0.001		<0.001	
**NO×10^3^ (ng/**ml)							
Q1 (< 33.90)	643	Ref		Ref		Ref	
Q2 (33.90–54.90)	641	1.01 (0.75–1.35)	0.97	1.01 (0.75–1.35)	0.97	0.92 (0.69–1.24)	0.59
Q3 (54.91–81.24)	647	0.89 (0.64–1.24)	0.50	0.89 (0.64–1.24)	0.50	0.79 (0.57–1.08)	0.14
Q4 (> 81.24)	645	0.90 (0.59–1.36)	0.61	0.90 (0.60–1.35)	0.61	0.82 (0.56–1.20)	0.31
*P* for trend		<0.001		<0.001		<0.001	
**CIO (ng/**ml)							
Q1 (< 2.44)	643	Ref		Ref		Ref	
Q2 (2.44–4.42)	645	0.99 (0.73–1.37)	0.99	0.99 (0.73–1.35)	0.96	0.94 (0.68–1.30)	0.71
Q3 (4.43–7.63)	644	0.84 (0.61–1.15)	0.28	0.83 (0.60–1.14)	0.25	0.78 (0.57–1.06)	0.12
Q4 (> 7.63)	644	0.76 (0.56–1.02)	0.07	0.74 (0.56–1.00)	0.05	0.70 (0.53–0.93)	0.02
*P* for trend		<0.001		<0.001		<0.001	

Adjusted covariates: model 1 = urinary creatinine, age, gender, ratio of family income to poverty and race; model 2 = model 1 + usual energy intake; model 3 = model 2 + mutual adjustment of urinary CIO, urinary NO, and urinary SCN.

Abbreviations: CI, confidence interval; OR, Odd ratio, SCN, thiocyanate, CIO, perchlorate, NO, nitrate.

**TABLE 3:** Associations between urinary CIO, urinary NO, and urinary SCN and the risk of overweight/obesity in adolescents (*N* = 1871)

Urinary biomarkers	*N*	Model 1	*P*	Model 2	*P*	Model 3	*P*
	1871	OR (95%CI)		OR (95%CI)		OR (95%CI)	
**SCN (ng/**ml)							
Q1 (< 586.00)	467	Ref		Ref		Ref	
Q2 (586.00–1140.00)	464	1.55 (1.11–2.17)	0.01	1.58 (1.13–2.19)	0.008	1.69 (1.24–2.30)	0.002
Q3 (1140.01–1990.00)	471	2.24 (1.65–3.04)	<0.001	2.29 (1.68–3.11)	<0.001	2.57 (1.92–3.45)	<0.001
Q4 (> 1990.00)	469	2.59 (1.87–3.58)	<0.001	2.63 (1.91–3.63)	<0.001	3.03 (2.23–4.11)	<0.001
*P* for trend		<0.001		<0.001		<0.001	
**NO×10^3^ (ng/**ml)							
Q1 (< 32.55)	468	Ref		Ref		Ref	
Q2 (32.55–53.60)	467	1.42 (0.94–2.15)	0.10	1.41 (0.94–2.12)	0.10	1.35 (0.89–2.05)	0.17
Q3 (53.61–79.30)	468	1.43 (1.03–1.98)	0.04	1.43 (1.03–1.99)	0.04	1.32 (0.94–1.85)	0.11
Q4 (> 79.30)	468	1.28 (0.87–1.88)	0.22	1.26 (0.86–1.85)	0.24	1.14 (0.74–1.75)	0.55
*P* for trend		<0.001		<0.001		<0.001	
**CIO (ng/mL)**							
Q1 (< 1.94)	467	Ref		Ref		Ref	
Q2 (1.94–3.64)	467	1.10(0.81–1.49)	0.55	1.11(0.82–1.50)	0.52	1.10(0.79–1.51)	0.58
Q3 (3.65–6.19)	468	1.15(0.83–1.06)	0.42	1.16(0.83–1.61)	0.39	1.18(0.82–1.68)	0.38
Q4 (> 6.19)	469	1.39(0.97–1.97)	0.07	1.41(0.98–2.03)	0.07	1.42(0.96–2.09)	0.08
*P* for trend		<0.001		<0.001		<0.001	

Adjusted covariates: model 1 = urinary creatinine, age, gender, ratio of family income to poverty and race; model 2 = model 1 + physical activity, usual energy intake; model 3 = model 2 + mutual adjustment of urinary CIO, urinary NO, and urinary SCN.

Abbreviations: CI, confidence interval; OR, Odd ratio; SCN, thiocyanate; CIO, perchlorate, NO, nitrate.

**TABLE 4:** Associations between urinary CIO, urinary NO, and urinary SCN and the risk of central obesity in children (*N* = 2576)

Urinary biomarkers	*N*	Model 1	*P*	Model 2	*P*	Model 3	*P*
	2576	OR (95%CI)		OR (95%CI)		OR (95%CI)	
**SCN (ng/**ml)							
Q1 (< 582.75)	644	Ref		Ref		Ref	
Q2 (582.75–1030.00)	632	1.06 (0.76–1.48)	0.72	1.06 (0.77–1.48)	0.71	1.08 (0.78–1.50)	0.65
Q3 (1030.01–1680.00)	648	1.16 (0.82–1.65)	0.40	1.17 (0.82–1.66)	0.39	1.18 (0.83–1.68)	0.36
Q4 (> 1680.00)	652	1.82 (1.24–2.70)	0.003	1.83 (1.24–2.70)	0.003	1.87 (1.27–2.76)	0.002
*P* for trend		<0.001		<0.001		<0.001	
**NO×10^3^ (ng/**ml)							
Q1 (< 33.90)	643	Ref		Ref		Ref	
Q2 (33.90–54.90)	641	1.13 (0.79–1.61)	0.49	1.13 (0.79–1.62)	0.49	1.05 (0.74–1.50)	0.78
Q3 (54.91–81.24)	647	1.14 (0.79–1.66)	0.48	1.14 (0.79–1.66)	0.48	1.03 (0.72–1.47)	0.89
Q4 (> 81.24)	643	0.98 (0.66–1.46)	0.92	0.98 (0.66–1.46)	0.92	0.90 (0.62–1.31)	0.58
*P* for trend		<0.001		<0.001		<0.001	
**CIO (ng/**ml)							
Q1 (< 2.44)	643	Ref		Ref		Ref	
Q2 (2.44–4.42)	645	1.19 (0.85–1.68)	0.32	1.19 (0.85–1.68)	0.31	1.15 (0.81–1.63)	0.43
Q3 (4.43–7.63)	644	1.00 (0.72–1.39)	0.99	1.00 (0.72–1.40)	0.99	0.97 (0.70–1.33)	0.83
Q4 (> 7.63)	644	0.97 (0.68–1.38)	0.87	0.97 (0.68–1.38)	0.87	0.94 (0.66–1.35)	0.75
*P* for trend		<0.001		<0.001		<0.001	

Adjusted covariates: model 1 = urinary creatinine, age, gender, ratio of family income to poverty and race; model 2 = model 1 + usual energy intake; model 3 = model 2 + mutual adjustment of urinary CIO, urinary NO, and urinary SCN.

Abbreviations: CI, confidence interval; OR, Odd ratio; SCN, thiocyanate; CIO, perchlorate; NO, nitrate.

**TABLE 5:** Associations between urinary CIO, urinary NO, and urinary SCN and the risk of central obesity in adolescents (*N* = 1871)

Urinary biomarkers	*N*	Model 1	*P*	Model 2	*P*	Model 3	*P*
	1871	OR (95%CI)		OR (95%CI)		OR (95%CI)	
**SCN (ng/**ml)							
Q1 (< 586.00)	467	Ref		Ref		Ref	
Q2 (586.00–1140.00)	464	1.23 (0.88–1.72)	0.22	1.25 (0.90–1.74)	0.18	1.31 (0.96–1.80)	0.10
Q3 (1140.01–1990.00)	471	1.63 (1.22–2.18)	0.002	1.65 (1.23–2.22)	0.001	1.81 (1.34–2.42)	<0.001
Q4 (> 1990.00)	469	1.23 (1.58–3.00)	<0.001	2.22 (1.62–3.03)	<0.001	2.45 (1.78–3.37)	<0.001
*P* for trend		<0.001		<0.001		<0.001	
**NO×10^3^ (ng/**ml)							
Q1 (< 32.55)	468	Ref		Ref		Ref	
Q2 (32.55–53.60)	467	1.18 (0.78–1.79)	0.44	1.17 (0.77–1.77)	0.47	1.11 (0.73–1.69)	0.62
Q3 (53.61–79.30)	468	1.29 (0.92–1.80)	0.14	1.29 (0.92–1.81)	0.14	1.18 (0.85–1.65)	0.33
Q4 (> 79.30)	468	1.13 (0.75–1.72)	0.55	1.12 (0.74–1.69)	0.59	0.99 (0.63–1.56)	0.98
*P* for trend		<0.001		<0.001		<0.001	
**CIO (ng/**ml)							
Q1(< 1.94)	467	Ref		Ref		Ref	
Q2(1.94–3.64)	467	1.00 (0.75–1.32)	0.99	0.99 (0.75–1.33)	0.97	1.01 (0.74–1.36)	0.96
Q3(3.65–6.19)	468	1.06 (0.74–1.53)	0.73	1.08 (0.75–1.55)	0.69	1.12 (0.76–1.64)	0.58
Q4 (> 6.19)	469	1.27 (0.91–1.77)	0.16	1.29 (0.93–1.82)	0.14	1.35 (0.94–1.94)	0.11
*P* for trend		<0.001		<0.001		<0.001	

Adjusted covariates: model 1 = urinary creatinine, age, gender, ratio of family income to poverty and race; model 2 = model 1 + physical activity, usual energy intake; model 3 = model 2 + mutual adjustment of urinary CIO, urinary NO, and urinary SCN.

Abbreviations: CI, confidence interval; OR, Odd ratio; SCN, thiocyanate; CIO, perchlorate; NO, nitrate.

**FIGURE 1:**
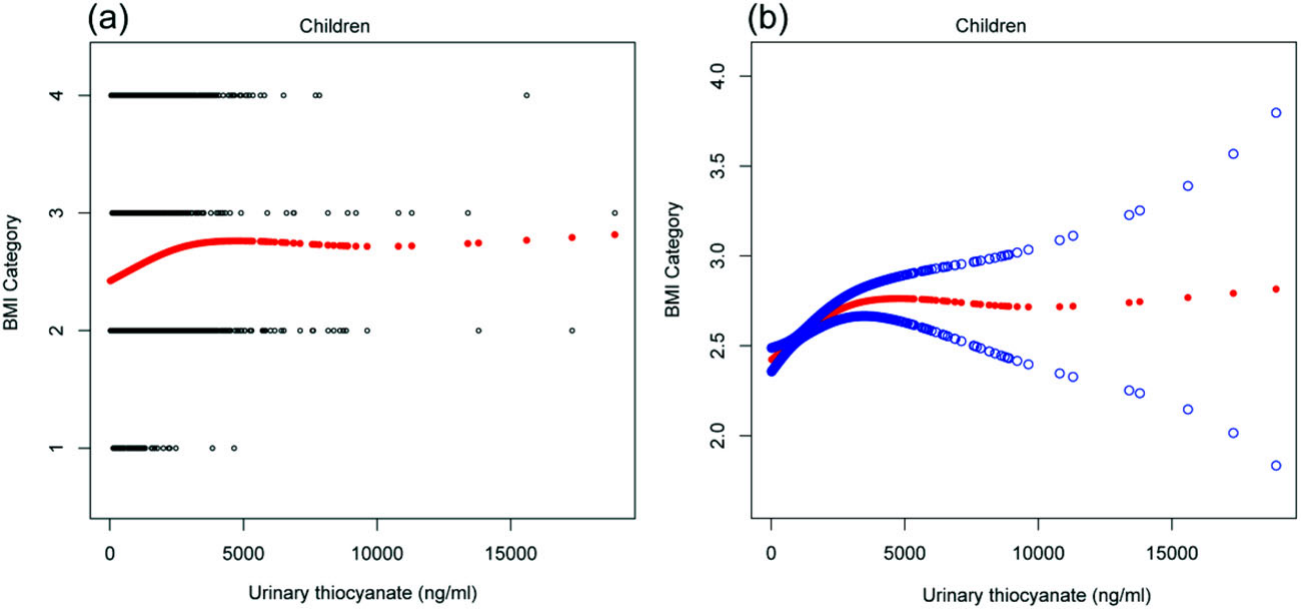
(a) The scatter curve of the relationship between urinary thiocyanate (SCN) level, and body mass index (BMI) category after adjusting for urinary creatinine, age, gender, ratio of family income to poverty, race, usual energy intake, urinary perchlorate (CIO) level, and urinary nitrate (NO) level in children. The blue and black lines indicate 95% CI and red lines indicate a smooth curve fitting line. (b) The smooth curve of the relationship between urinary SCN level and BMI category after adjusting for urinary creatinine, age, gender, race, ratio of family income to poverty, usual energy intake, urinary CIO, and urinary NO in children.

**FIGURE 2:**
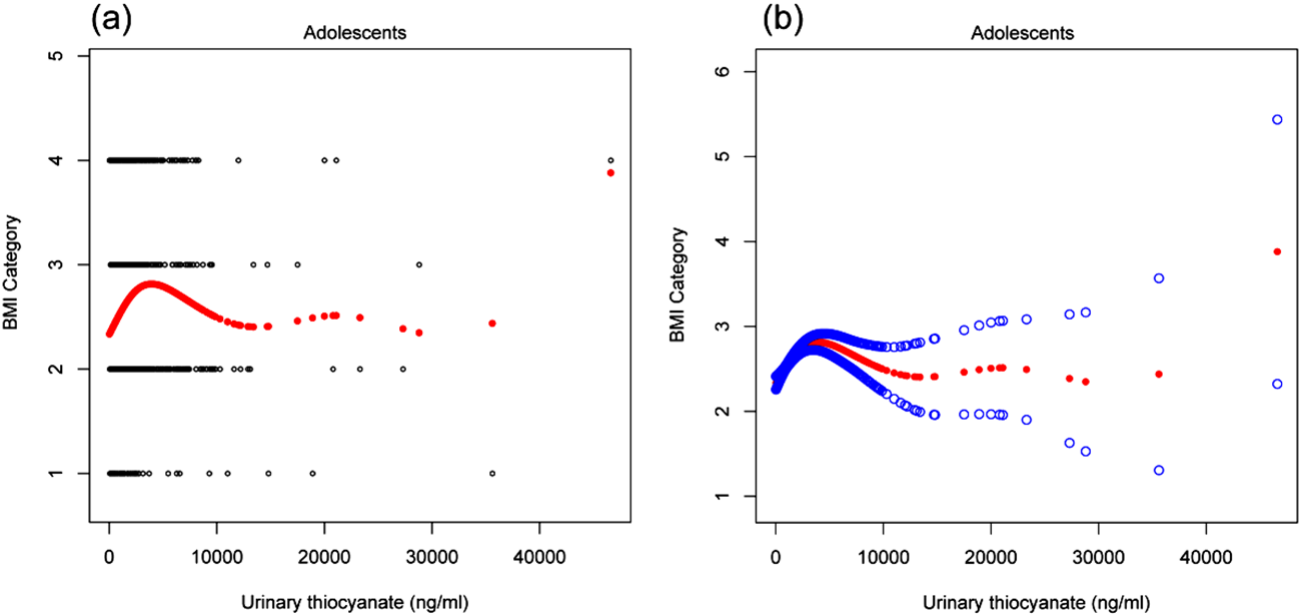
(a) The scatter curve of the relationship between urinary thiocyanate (SCN) level and body mass index (BMI) category after adjusting for urinary creatinine, age, gender, ratio of family income to poverty, race, physical activity, usual energy intake, urinary perchlorate (CIO) level, and urinary nitrate (NO) level in adolescents. The blue and black lines indicate 95% CI and red lines indicate a smooth curve fitting line. (b) The smooth curve of the relationship between urinary SCN level and BMI category after adjusting for urinary creatinine, age, gender, ratio of family income to poverty, race, physical activity, usual energy intake, urinary CIO level, and urinary NO level in adolescents.

We found that the relationship between BMI category and urinary SCN was non‐linear after adjusting for urinary creatinine, physical activity (aged 12–19 years), age, ratio of family income to poverty, race, sex, total energy intake, urinary CIO level, and urinary NO level in children and adolescents (Figures 1 and 2, respectively).

As shown in Table 6, the inflection point was 1970 ng/ml in children and 1940 ng/ml in adolescents. In children, there was a saturation effect between BMI category and urinary SCN level. On the left of the inflection point (SCN < 1970 ng/ml), the β, 95% CI, and *P* values were 0.0002, 0.0001–0.0002, and < 0.001, respectively. BMI category increased with an increase in urinary SCN level. When SCN level was ≥1970 ng/ml, BMI category stopped increasing. In adolescents, we found a threshold effect between BMI category and urinary SCN level. On the left of the inflection point (SCN < 1940 ng/ml), the β, 95% CI, and *P* values were 0.0002, 0.0002–0.0003 and < 0.0001, respectively. BMI category increased with an increase in urinary SCN level. On the right of the inflection point (SCN ≥ 1940 ng/ml), BMI category decreased with an increase in urinary SCN level.

**TABLE 6:** Association between urinary SCN and BMI category by segmented regression and log‐likelihood ratio test in children (*N* = 2576) and adolescents (*N* = 1871)

	Crude β (95% CI) *P*‐value	Adjusted^a^ β (95% CI) *P*‐value
**Children**		
SCN < 1970 ng/ml	0.0002 (0.0001, 0.0003) < 0.001	0.0002 (0.0001, 0.0002) < 0.001
SCN ≥ 1970 ng/ml	0.0000 (−0.0000, 0.0000) 0.36	0.0000 (−0.0000, 0.0000) 0.44
**Adolescents**		
SCN < 1940 ng/ml	0.0002 (0.0001, 0.0003) < 0.001	0.0002 (0.0002, 0.0003) < 0.001
SCN ≥ 1940 ng/ml	−0.0000 (−0.0000, 0.0000) 0.30	−0.0000 (−0.0000, 0.0000) 0.31

^a^ Adjusted for urinary creatinine, age, gender, race, ratio of family income to poverty, physical activity (adolescents), usual energy intake and mutual adjustment of urinary CIO and urinary NO.

Abbreviations: CI, confidence interval; SCN, thiocyanate; CIO, perchlorate; NO, nitrate.

## 4 DISCUSSION

In our study, we demonstrated, for the first time, that urinary SCN was positively associated with overweight/obesity and central obesity in children and adolescents, regardless of age, sex, physical activity (in adolescents), usual energy intake, urinary creatinine, CIO, and NO levels. We found a saturation effect in the children and a threshold effect in the adolescents between BMI category and urinary SCN level. In addition, higher exposure to CIO is associated with a lower risk of obesity in children

Obesity is one of the most important public health challenges of the 21st century, as declared by the World Health Organization. Therefore, preventing obesity is an important public health undertaking. This is especially important because of overweight and obese children and adolescents, who are susceptible to maintaining obesity into adulthood and developing severe chronic diseases such as diabetes and cardiovascular disease at a younger age. Although several factors have been proven to be involved in the ‘obesity pandemic’, in recent years, more focus has been on exposure to specific environmental pollutants, the obesogens (Sousa Ana et al., 2022).

Obesogens can disrupt the endocrine system, which regulates metabolism and weight gain (Grün et al., 2006). Throughout our lifetime, chronic exposure to obesogens is likely the primary cause of obesity and metabolic disorders (Trasande et al., 2016). Some obesogens (e.g., bisphenols and phthalates) are lipophilic, accumulating in the adipose tissue of obese women and changing their chromatin organization and structure to influence the development of obesity in their offspring (Mohanto et al., 2021). Microplastics can induce oxidative stress and affect adipocyte differentiation following accumulation in liver and kidney, altering the energy balance and lipid metabolism (Kannan et al., 2021). Furthermore, additives and monomers cause indirect toxicity for humans by promoting adipogenesis and lipid accumulation (Mohanto et al., 2021). A previous study demonstrated that chlorophenol, another environmental pollutant, was positively associated with central obesity in Korean girls. Humans are exposed to this pollutant through the consumption of contaminated foods and drinking water, dermal contact, and inhalation of polluted air (Moon et al., 2021). Therefore, investigating the associations between exposures to these chemicals and overweight/obesity and central obesity is pivotal to the reduction of obesity incidence.

The mean values of urinary SCN in children were 840 ng/ml, 9470 ng/ml, 4000 ng/ml, and 420 ng/ml in India, Mozambique, Turkey, and Iran, respectively (Chandra et al., 2008; Cliff et al., 1999; Erdoğan et al., 2001; Keshteli et al., 2010). In comparison, the mean value of urinary SCN in children was moderate in the U.S. The mean value of urinary CIO in children was 11.7 ng/ml in Kuwaiti (Alomirah et al., 2016), while the mean value of urinary CIO in children from the U.S. was lower. A study reported that adolescents of different ethnicities have different urinary CIO, SCN, and NO levels (Liu et al., 2017). Our subgroup analysis showed that there was no significant association between urinary SCN level and obesity in ‘other race’ children and ‘other Hispanic’ adolescents. The ethnic differences may be due to differences in diet, exposure level, and metabolism. Several previous studies have revealed relationships between CIO, NO, and SCN levels and adverse effects such as death risk from cancer in smokers, allergic symptoms, cancer risk, and pulmonary disorders (Zhu et al., 2021; Shiue et al., 2015; Wang et al., 2001). The results from ecologic, experimental, and observational studies have shown divergent associations of exposure to SCN, NO, and CIO with thyroid hormone functions in different population groups, including adults, adolescents, pregnant women, and infants. The association between these three chemicals and obesity remains mixed. A previous study has shown that higher exposure to NO was associated with a lower risk of obesity and higher exposure to SCN was associated with a higher risk of obesity in adults (Zhu et al., 2019). In contrast, a study that enrolled 940 American girls (aged 6–8 years) suggested that higher SCN, NO, and CIO exposures were associated with lower waist circumference and BMI (Mervish et al., 2016). In our study, we found that higher exposure to SCN was associated with a higher risk of overweight/obesity and central obesity both in children and adolescents. There may be a saturation effect in children and a threshold effect in adolescents due to a compensatory response to SCN exposure in the association between urinary SCN level and overweight/obesity. This may account for the discrepancy with the results of previous studies. We found that urinary CIO level was associated negatively with obesity in children, showing that children were vulnerable to CIO exposure.

SCN, NO, and CIO compete with iodine for uptake into the thyroid by the sodium iodide symporter. Several studies have demonstrated that thyroid stimulating hormone (TSH) is associated positively with weight gain in children (Thiagarajan et al., 2020; Wang et al., 2020). TSH can stimulate adipogenesis and fat accumulation directly (Zhang et al., 2019). The association between TSH and SCN remains contradictory. In women with urinary iodine levels above 100 μg/L, urinary SCN level was associated negatively with serum TSH (Blount et al., 2006). However, a previous study found that SCN exposure was associated positively with TSH levels in infants (Cao et al., 2010). There may be a difference in the association between TSH and SCN between adults and children. We found that, when urinary SCN was beyond the threshold, the risk of obesity decreased in the adolescents, which may account for this difference. Moreover, SCN level is a major exposure marker of cyanide (Bhandari et al., 2014). A study using an animal model showed that cyanide might have influenced body weight changes (De Sousa et al., 2014), indicating that SCN may play a role in weight gain through the effect of TSH and cyanide. While we found that only CIO was associated negatively with obesity in children, another study indicated that CIO exposure was associated positively with T4 in infants (Blount et al., 2006). T4 may lead to an increase in energy expenditure, causing weight loss (McAninch et al., 2014). These are potential mechanisms by which CIO and SCN may influence overweight/obesity and central obesity.

The strengths of our study included the large sample size, use of a representative, multiracial population, and the involvement of smooth curve fitting and segmented regression and log‐likelihood ratio tests in determining whether a threshold existed. However, there were also several limitations in our study. One of the limitations was the temporality and residual confounding of this cross‐sectional study. Another limitation was that the single measurement of these three chemicals in urine samples may not have reflected long‐term exposure among children and adolescents. Despite these limitations, our study provided insight into the association of CIO, NO, and SCN levels with overweight, obesity in children and adolescents.

The findings of our study suggested that urinary SCN level was associated positively with overweight/obesity and central obesity in both children and adolescents. In children, we observed a saturation effect, which indicated that, when the urinary SCN level increased beyond the inflection point, the risk of obesity stopped increasing. In adolescents, we observed a threshold effect, which indicated that, when the urinary SCN level increased beyond the inflection point, the risk of obesity decreased. We found that only urinary CIO level was associated negatively with overweight/obesity in children. However, no significant associations were observed for urinary NO. Therefore, routine tests for these chemicals in water and food and routine tests for these chemicals in the serum and urine of obese children and adolescents are recommended to reduce the incidence of obesity in children and adolescents. Further studies to determine the underlying mechanisms of these associations between exposure to these chemicals and overweight/obesity and central obesity are warranted.

## CONFLICTS OF INTEREST

The authors declare no conflict of interest
